# Delayed anastomotic leakage following laparoscopic intersphincteric resection for lower rectal cancer: report of four cases and literature review

**DOI:** 10.1186/s12957-017-1208-2

**Published:** 2017-08-01

**Authors:** Masayoshi Iwamoto, Kenji Kawada, Koya Hida, Suguru Hasegawa, Yoshiharu Sakai

**Affiliations:** 1 Department of Surgery, Kyoto University Graduate School of Medicine, 54 Shogoin-Kawara-cho, Sakyo-ku, Kyoto, 606-8507 Japan; 2 Department of Surgery, National Hospital Organization Himeji Medical Center, Himeji, Japan; 3 Department of Gastroenterological Surgery, Faculty of Medicine, Fukuoka University, Fukuoka, Japan

**Keywords:** Delayed anastomotic leakage, Intersphincteric resection, Rectal cancer, Surgery

## Abstract

**Background:**

Anastomotic leakage (AL) is one of the most dreadful postoperative complications because it can result in increased morbidity and mortality as well as poorer long-term prognosis. Although most studies of AL limited their investigation time to a period of 30 days postoperatively, only a few studies have shown that AL can occur after that period. Here, we report four patients of rectal cancer with delayed AL following laparoscopic intersphincteric resection (ISR) and conduct a literature review on delayed AL.

**Case presentation:**

Case 1 was a 67-year-old male who underwent laparoscopic partial ISR in July 2009. Although the patient was asymptomatic, an anastomotic-urethral fistula was observed 57 months after ISR. Case 2 was a 44-year-old female who underwent laparoscopic partial ISR in July 2008. She presented with discharge of gas and feces from her vagina, and an anastomotic-vaginal fistula was observed 14 months after ISR. Case 3 was a 74-year-old man who underwent laparoscopic partial ISR in August 2007. He presented with pneumaturia and fecaluria, and an anastomotic-urethral fistula was observed 4 months after ISR. Case 4 was a 68-year-old woman who underwent laparoscopic subtotal ISR for rectal cancer in February 2013 and partial hepatic resection for liver metastases in March 2013. She presented with anal pain and purulent perineal discharge, and an anastomotic-perineal fistula was observed 9 months after ISR. All four cases presented with fistula formation and required reoperation (establishment of a diverting ileostomy).

**Conclusions:**

Since delayed AL is not a rare postoperative complication, surgeons need to provide long-term follow-up and remain alert to the possible development of delayed AL.

## Background

The introduction of intersphincteric resection (ISR) is one of the recent advances in the surgical treatment of lower rectal cancer. ISR is a surgical technique to preserve sphincter function that was first described by Schiessel et al. [1]. Several studies have demonstrated the acceptable outcomes of ISR in terms of morbidity, oncologic safety, and postoperative anal functions, and ISR has been proposed as an alternative to abdominoperineal resection (APR) for selected patients with lower rectal cancer [2–5].

Anastomotic leakage (AL) is one of the most dreadful postoperative complications of colorectal cancer because it can result in increased morbidity and mortality as well as poorer long-term prognosis. Reported incidence rates of colorectal AL vary between 3 and 20% [6–8]. Although most studies of AL have limited their investigation time to a period of postoperative 30 days, some studies have shown that AL can occur more than 30 days postoperatively [9–14]. To date, there is no consensus as to an exact definition of delayed AL, and there is little information on delayed AL. Delayed AL has been defined as AL diagnosed after hospital discharge [11] or as AL diagnosed more than 30 days postoperatively [12–14]. Shin et al. proposed the following criteria for delayed AL: (1) AL was detected more than 3 weeks postoperatively, (2) a normal diet and defecatory function was resumed within 1 week of surgery, (3) AL developed without the occurrence of any signs or symptoms of peritonitis within the postoperative 3 weeks, and (4) no local recurrence developed during the follow-up period [10].

Here, we reported four cases of patients with delayed AL following laparoscopic ISR and conducted a review of the literature about delayed AL following colorectal surgery. To the best of our knowledge, this is the first report of delayed AL following ISR.

## Case presentation

### Case 1

**Fig. 1:**
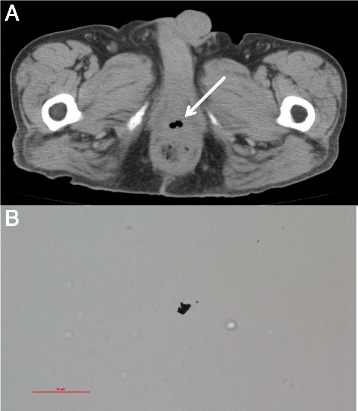
**a** Case 1. CT scan revealed the presence of a small amount of extraluminal air between the prostate and rectum, adjacent to the coloanal anastomosis (*arrow*). **b** Case 1. Orally administered medicinal charcoal was detected in the urine

A 67-year-old male who was diagnosed with lower rectal cancer underwent laparoscopic partial ISR with creation of a diverting ileostomy in July 2009. The pathological analysis indicated that the tumor staging was stage I (pT2N0M0 according to the 7th edition UICC) with negative resection margins. He did not receive adjuvant chemotherapy or radiotherapy. The ileostomy was reversed in August 2009, and he did well clinically for more than 4 years. In April 2014, although he was asymptomatic, laboratory blood tests showed signs of mild inflammation (WBC, 11,200 /μL; C-reactive protein, 2.2 mg/dL). Computed tomography (CT) scan revealed the presence of a small amount of extraluminal air between the prostate and rectum, adjacent to the coloanal anastomosis (Fig. 1a), which had not been observed on the previous follow-up CT scans. Both colonoscopy and cystoscopy were unable to detect a recurrent tumor or fistula on the anastomosis, but the presence of an anastomotic-urethral fistula was confirmed when orally administered medicinal charcoal was detected in the urine (Fig. 1b). He underwent reestablishment of an ileostomy, which has not been reversed for more than 1 year because the fistula remains.

### Case 2

**Fig. 2:**
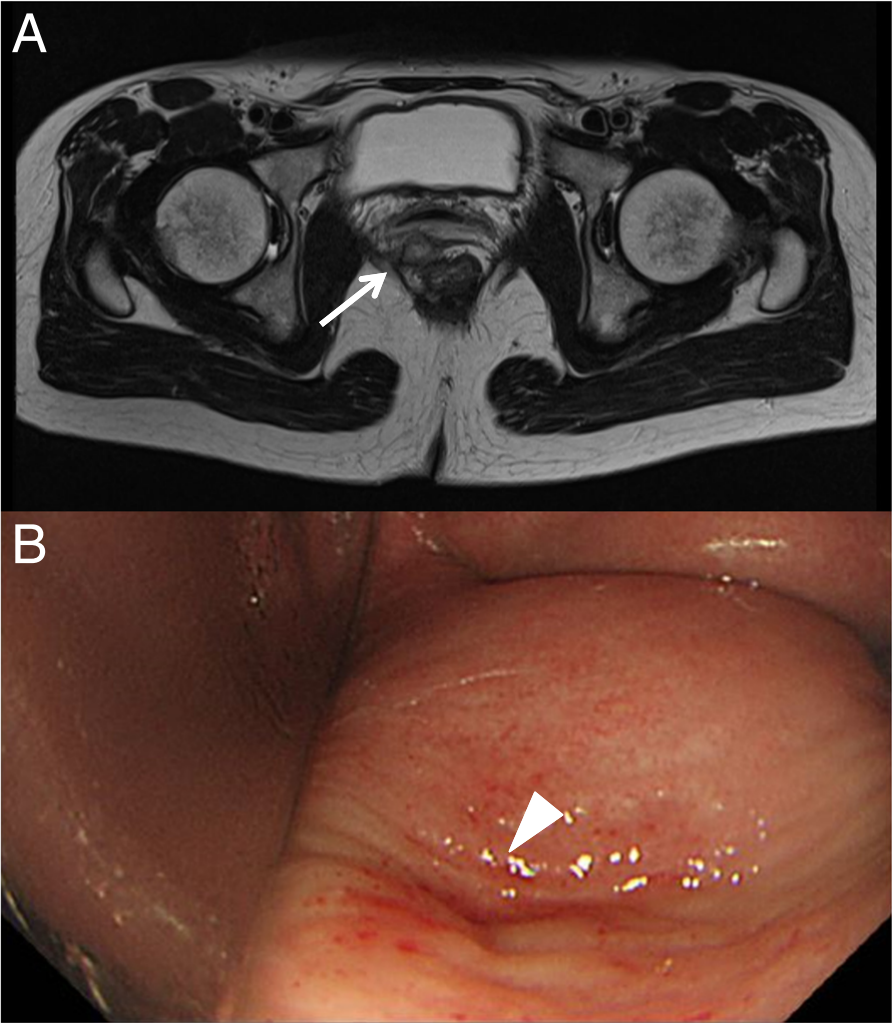
**a** Case 2. MRI revealed that an anastomotic-vaginal fistula existed at the right anterior side of the coloanal anastomosis (*arrow*). **b** Case 2. Colposcopy revealed a pin-hole fistula on the posterior wall of the vagina (*arrow head*)

A 44-year-old female went to a hospital in December 2007 complaining of anal bleeding. She was diagnosed with internal hemorrhoids, and she was treated using an aluminum potassium sulfate and tannic acid (ALTA) injection. However, her anal bleeding continued and she was admitted to our hospital because lower rectal cancer was additionally detected by colonoscopy. She underwent laparoscopic partial ISR with creation of a diverting ileostomy in July 2008, and the pathological analysis indicated that the tumor staging was stage I (pT2N0M0) with negative resection margins. She received adjuvant chemotherapy (UFT and leucovorin) for 12 months, and the ileostomy was reversed in November 2008. In September 2009, 14 months after ISR, she suddenly experienced a discharge of gas and feces from her vagina. CT and magnetic resonance imaging (MRI) revealed that an anastomotic-vaginal fistula existed at the right anterior side of the coloanal anastomosis (Fig. 2a). Colposcopy revealed a pin-hole fistula on the posterior wall of the vagina (Fig. 2b), and the biopsy from the fistula was not indicative of malignancy. She underwent reestablishment of an ileostomy, and subsequent follow-up CT and MRI showed no local recurrence for more than 1 year. The ileostomy was reversed in October 2010, and she has been alive without recurrence for more than 6 years.

### Case 3

**Fig. 3:**
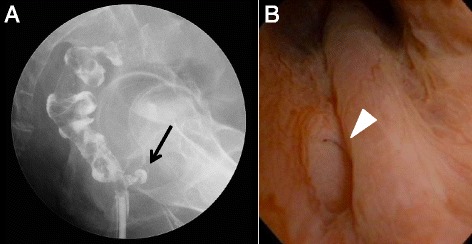
**a** Case 3. Contrast enema revealed that an anastomotic-urethral fistula existed (*arrow*). **b** Case 3. Cystoscopy identified that it was located at the distal edge of the prostate (*arrow head*)

A 74-year-old man who was diagnosed with lower rectal cancer underwent laparoscopic partial ISR in August 2007. The pathological analysis indicated that the tumor staging was stage 0 (pTisN0M0) with negative resection margins. In December 2007, 4 months after ISR, he presented with pneumaturia and fecaluria. Contrast enema revealed that an anastomotic-urethral fistula existed (Fig. 3a), and cystoscopy identified that it was located at the distal edge of the prostate (Fig. 3b). He underwent reestablishment of an ileostomy. The ileostomy was then reversed in January 2010 after the closure of anastomotic-urethral fistula was confirmed. He has been alive without recurrence for more than 7 years.

### Case 4

**Fig. 4:**
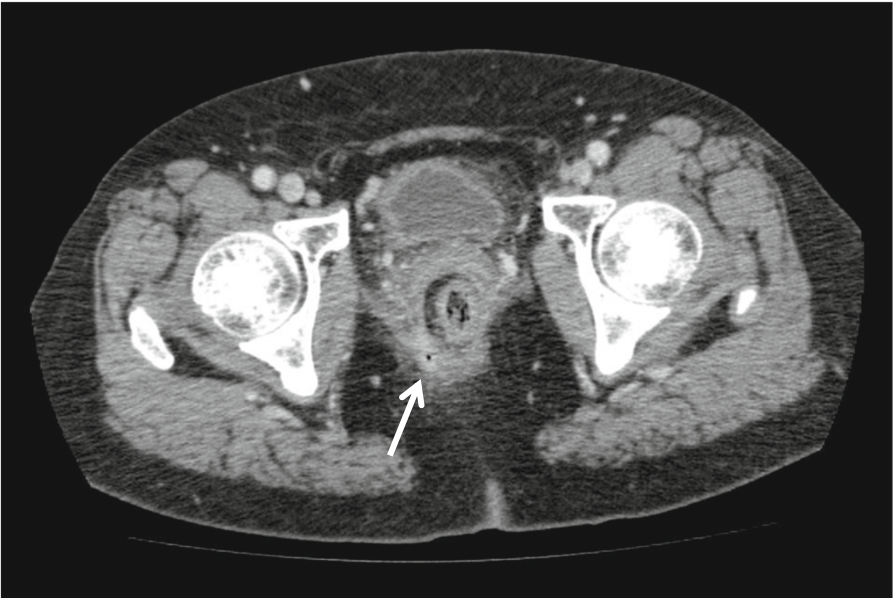
Case 4. CT scan revealed a small amount of extraluminal air existed along an anastomotic-perineal fistula (*arrow*)

A 68-year-old woman who had undergone right hepatic lobectomy for hilar cholangiocarcinoma at the age of 60 years was diagnosed with lower rectal cancer with two liver metastases in February 2013. She underwent laparoscopic subtotal ISR for rectal cancer, and then, 1 month later, she underwent partial hepatic resection for liver metastases. The pathological findings indicated that the tumor staging was stage IV (pT3N0M1) with negative resection margins and that curative resection could be achieved. In December 2013, 9 months after ISR, she presented with anal pain and purulent perineal discharge. Colonoscopy and CT scan revealed that an anastomotic-perineal fistula existed on the right anterior side of the coloanal anastomosis (Fig. 4), and then, she underwent reestablishment of an ileostomy. In February 2014, CT scan showed several metastases in the lung and she has received systemic chemotherapy for more than 2 years.

## Discussion

The gold standard of surgical technique for rectal cancer is total mesorectal excision (TME), which results in improved survival and reduced local recurrence. In recent years, ISR for lower rectal cancer has been performed in selected patients as an alternative to APR. ISR involves the transanal division of the distal rectum, removal of part or all of the internal anal sphincter, and restoration of bowel continuity by performing handsewn coloanal anastomosis. By performing TME simultaneously, ISR is thought to afford adequate oncological resection margins while preserving sphincter function [2–5]. In addition, laparoscopic ISR is touted as a minimally invasive technique [5].

AL is one of the most serious complications following colorectal surgery. While AL is commonly believed to occur within 30 days postoperatively, recent studies have shown that AL can occur beyond the 30-day postoperative period. Here, we reported four cases of patients with delayed AL following laparoscopic ISR. In all four cases, AL occurred more than 1 month after surgery: postoperative months 4, 9, 14, and 57. The literature search yielded only a few English-language publications on delayed AL following colorectal surgery (Table 1). Importantly, there was no previous report on delayed AL following ISR. The incidence rate of delayed AL was reported to be relatively high (i.e., 0.3–4.3%), approximately one third of all AL cases [9–14]. A retrospective review of our prospective database from July 2005 to June 2015 suggested that a total of 41 rectal cancer patients underwent laparoscopic ISR at our institution and that the incidence rate of delayed AL was 9.8% (4/41), whereas that of early AL (within less than 30 days postoperatively) was 0% (0/41). Preoperative chemoradiotherapy or chemotherapy was not performed in these 4 patients with delayed AL, indicating delayed AL was not associated with preoperative chemoradiotherapy or chemotherapy in this series (Table 2). In addition, no correlation was found in terms of sex, UICC-TNM stage, and lateral lymph node dissection (Table 2). In that same period, 179 patients with rectal cancer underwent laparoscopic low anterior resection (LAR) with double stapling technique anastomosis at our institution. Regarding laparoscopic LAR, early AL occurred in 23 patients (23/179: 12.8%), while delayed AL did not occur at all. These findings suggest that delayed AL cannot actually be considered a rare complication; therefore, surgeons should provide long-term follow-up and remain alert to the possible development of delayed AL.

**Table 1:** Description of cases with delayed anastomotic leakage (AL) following colorectal cancer

Author	Number of delayed AL	Timing of delayed AL	Operation method	Fistula formation (*n*, %)	Needed reoperation (*n*, %)	Risk factors of delayed AL
Hyman et al. [9]	4	More than POD 30	ND	ND	ND	ND
Shin et al. [10]	24	More than POD 21	AR	10/24 (42%)	24/24 (100%)	Female, low-level anastomosis preoperative chemoradiation
Floodeen et al. [11]	18	After hospital discharge	LAR	6/18 (33%)	ND	Female, lower BMI, lower operation time, lower operative bleeding
Morks et al. [12]	9	More than POD 30	LAR	2/9 (22%)	4/9 (44%)	Preoperative radiation
Tan et al. [13]	6	More than POD 30	LAR, RH	6/6 (100%)	6/6 (100%)	Younger age, smoking, neoadjuvant therapy
Lim et al. [14]	56	More than POD 30	LAR	26/56 (46%)	31/56 (55%)	Preoperative radiation
Our cases	4	More than POD 30	ISR	4/4 (100%)	4/4 (100%)	

*ND* not described, *POD* postoperative days, *AR* anterior resection, *LAR* low anterior resection, *RH* right hemicolectomy, *ISR* intersphincteric resection

**Table 2:** Characteristics of patients following laparoscopic ISR (*n* = 41)

Characteristics	Delayed AL (+) (*n* = 4)	Delayed AL (−) (*n* = 37)
Sex		
Male	2	24
Female	2	13
UICC-TNM stage		
0	1	3
I	1	14
II	0	3
III	1	13
IV	1	4
Preoperative treatment		
Chemoradiotherapy	0	3
Chemotherapy	0	7
No	4	27
Lateral lymph node dissection		
Yes	0	7
No	4	30

There is a lack of understanding as to whether or not delayed AL is different from early AL. Reported risk factors for early AL following rectal surgery are low level of anastomosis, male gender, and the presence of intraoperative adverse events [6–8], which may correlate to the degree of surgical difficulty. In terms of the timeframe in which delayed AL develops, delayed AL does not seem to be attributable to technical factors, but rather to other predisposing factors. According to the findings of previous reports, there were no obvious differences in patient characteristics and surgical factors between early AL and delayed AL [10–14]. The main difference may lie in the extent of leakage, i.e., more severe leakages give rise to symptoms earlier, whereas less severe leakages take longer to develop. The causes or predisposing factors associated with delayed AL have not yet been elucidated. Tan et al. reported that patients with delayed AL were much younger and more prone to present with fistulas compared to those with early AL, while no significant difference was found between the two groups in terms of other factors including gender, body mass index, smoking, hypertension, preoperative albumin, and duration of surgery [13]. Floodeen et al. reported that leakage from the anterior side of the circular stapler line was more common in patients with delayed AL than in those with early AL and that there was a larger proportion of an anastomotic-vaginal fistula in delayed AL [11]. Shin et al. reported that the independent risk factors for delayed AL were female gender, low-level anastomosis, and preoperative chemoradiation therapy and that the rate of anastomotic-vaginal fistula was relatively high (42%) in delayed AL [10]. Recently, Lim et al. reported that delayed AL following LAR was associated with preoperative radiotherapy, fistula formation, and the less frequent need for reoperation [14]. In the procedure of ISR, the perineal approach (i.e., intersphincteric dissection and coloanal anastomosis) is commonly performed by direct vision. However, the field of vision in the perineal approach is poor, especially at the anterior side, rendering it one of the most difficult parts of the ISR procedure. In our cases, all four patients with delayed AL presented with fistulas at the anterior side of the coloanal anastomosis: two anastomotic-urethral fistulas, one anastomotic-vaginal fistula, and one anastomotic-perineal fistula. These occurrences may be related to the technical difficulty caused by poor visualization of surgical field at the anterior side. To resolve this problem, a promising alternative approach can be transanal TME. The transanal approach in ISR can provide better surgical field especially at the anterior side, which may reduce the incidence of postoperative complications, such as delayed AL.

## Conclusions

We report four cases of delayed AL following laparoscopic ISR with a review of the literature. Delayed AL is not a rare postoperative complication, and therefore, surgeons should provide long-term follow-up and remain alert to the possible development of delayed AL.

## Abbreviations

AL: Anastomotic leakage
ALTA: Aluminum potassium sulfate and tannic acid
APR: Abdominoperineal resection
CT: Computed tomography
ISR: Intersphincteric resection
LAR: Low anterior resection
MRI: Magnetic resonance imaging
TME: Total mesorectal excision
